# Supplementary material for: Application of machine learning with MALDI-TOF MS for rapid differentiation between methicillin-susceptible and methicillin-resistant Staphylococcus aureus
Source: PLoS Comput Biol. 2026 May 5;22(5):e1013760. doi: 10.1371/journal.pcbi.1013760 (PMC13166928; doi:10.1371/journal.pcbi.1013760)
Supplement: S1 Table — (DOCX) [file pcbi.1013760.s001.docx]

| Year | MSSA | MRSA | Total |
| --- | --- | --- | --- |
| 2021 | 2911 | 3450 | 6361 |
| 2022 | 3039 | 3726 | 6765 |
| 2023 | 3356 | 4498 | 7854 |
| 2024 (Jan – May) | 1405 | 2102 | 3507 |
| Specimen Type | MSSA | MRSA | Total |
| Wound swab | 5633 | 3089 | 8722 |
| Sputum | 716 | 1472 | 2188 |
| Blood | 886 | 595 | 1481 |
| Urine | 593 | 694 | 1287 |
| Tissue | 596 | 261 | 857 |
| Pus | 531 | 207 | 738 |
| Miscellaneous | 1756 | 7458 | 9214 |
| Total | 13776 | 10711 | 24487 |

S1 Table. Base dataset characteristics.
