# Supplementary material for: Application of machine learning with MALDI-TOF MS for rapid differentiation between methicillin-susceptible and methicillin-resistant Staphylococcus aureus
Source: PLoS Comput Biol. 2026 May 5;22(5):e1013760. doi: 10.1371/journal.pcbi.1013760 (PMC13166928; doi:10.1371/journal.pcbi.1013760)
Supplement: S2 Table — (DOCX) [file pcbi.1013760.s002.docx]

| Dataset | MSSA | MRSA | Total | MRSA/MSSA ratio |
| --- | --- | --- | --- | --- |
| Training | 8580 | 11010 | 19590 | 1.283216783 |
| Validation | 2131 | 2766 | 4897 | 1.297982168 |
| Prospective | 1108 | 1867 | 2975 | 1.685018051 |
| External | 500 | 500 | 1000 | 1 |

S2 Table. Class distribution across datasets used in this study.
