# Supplementary figures and images for: Application of machine learning with MALDI-TOF MS for rapid differentiation between methicillin-susceptible and methicillin-resistant Staphylococcus aureus
Source: PLoS Comput Biol. 2026 May 5;22(5):e1013760. doi: 10.1371/journal.pcbi.1013760 (PMC13166928; doi:10.1371/journal.pcbi.1013760)

| 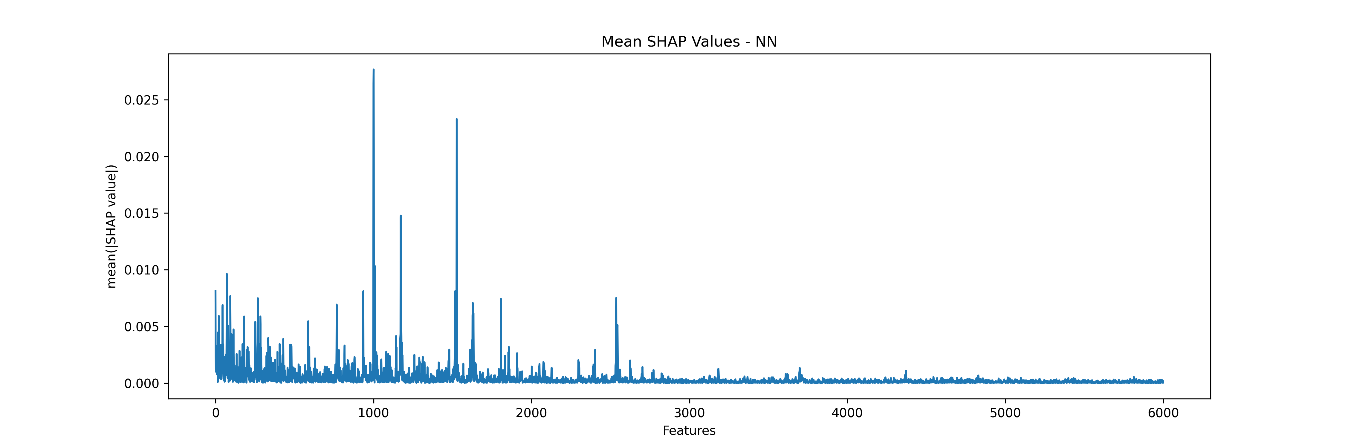 |
| --- |
| 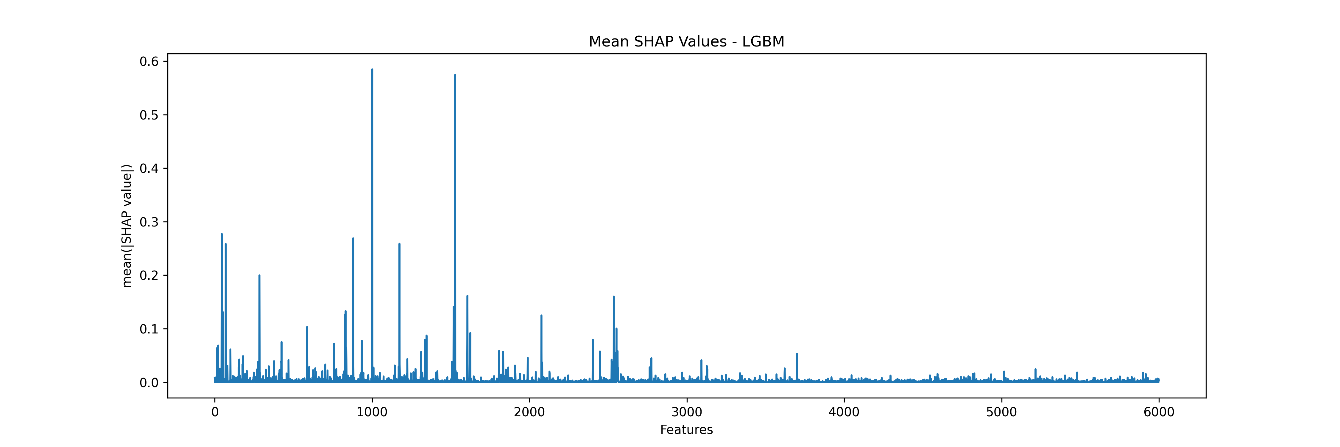 |
| 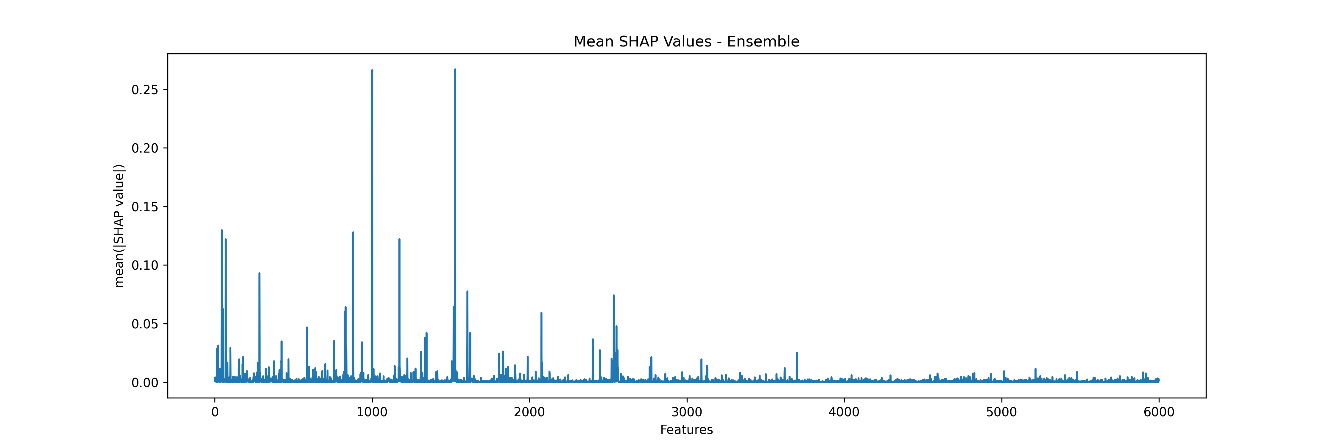 |

S1 Fig. Feature importance of the neural network, LightGBM, and ensemble model.

Supplement: S1 Fig — (DOCX) [file pcbi.1013760.s003.docx]
