# Supplementary material for: Application of machine learning with MALDI-TOF MS for rapid differentiation between methicillin-susceptible and methicillin-resistant Staphylococcus aureus
Source: PLoS Comput Biol. 2026 May 5;22(5):e1013760. doi: 10.1371/journal.pcbi.1013760 (PMC13166928; doi:10.1371/journal.pcbi.1013760)
Supplement: S2 Fig — A: feature 999–1002 (m/z 4997–5009), B: feature 1526–1528 (m/z 6578–6584), C: feature 1172–1175 (m/z 5516–5525). (DOCX) [file pcbi.1013760.s004.docx]

| A | 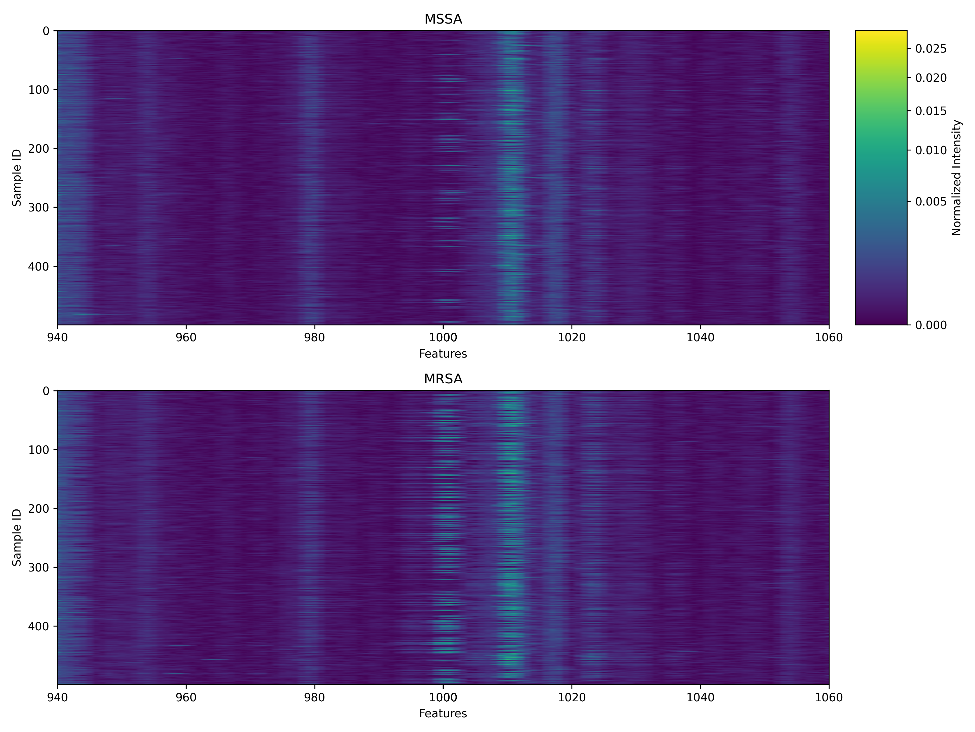 |
| --- | --- |
| B | 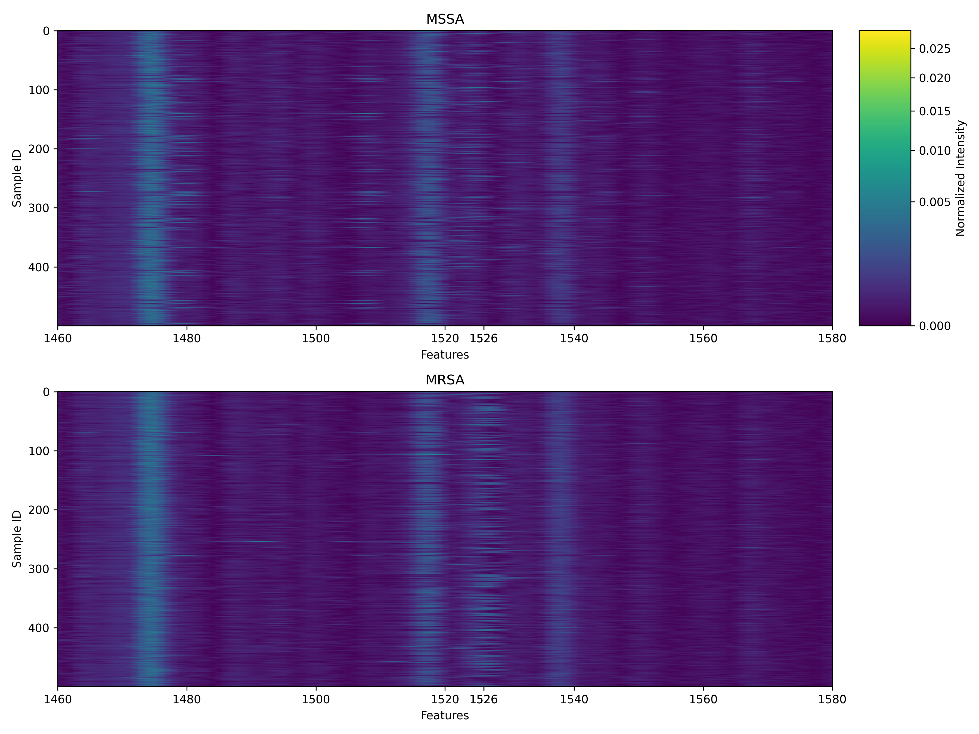 |
| C | 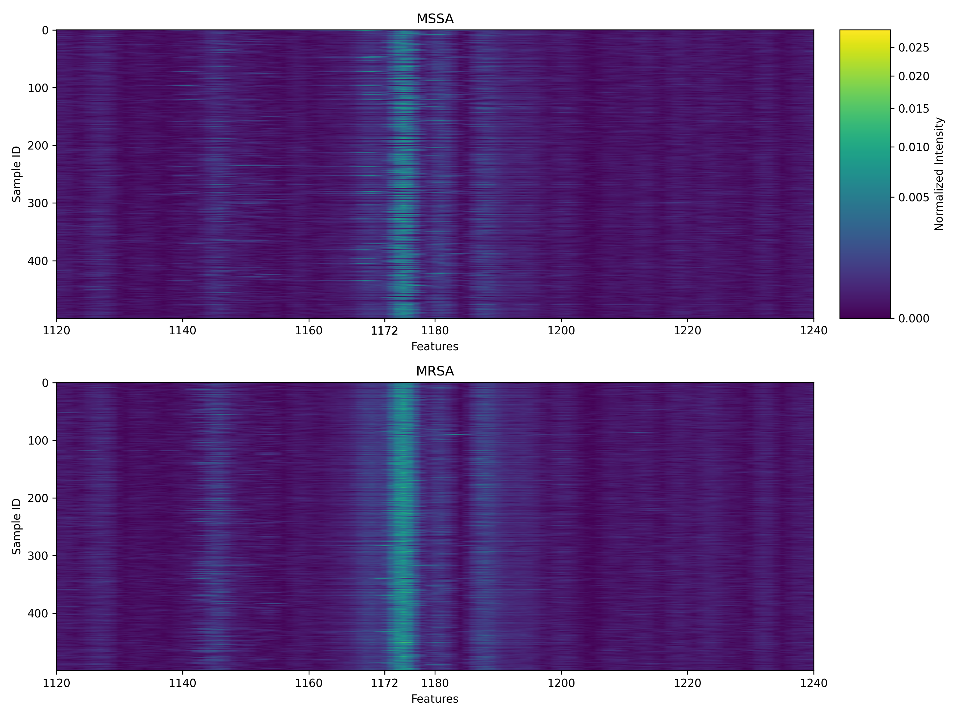 |

S2 Fig. Pseudogel plot using a 500-sample random subset of training data. A: feature 999-1002 (m/z 4997-5009), B: feature 1526-1528 (m/z 6578-6584), C: feature 1172-1175 (m/z 5516-5525).
