# Supplementary material for: Application of machine learning with MALDI-TOF MS for rapid differentiation between methicillin-susceptible and methicillin-resistant Staphylococcus aureus
Source: PLoS Comput Biol. 2026 May 5;22(5):e1013760. doi: 10.1371/journal.pcbi.1013760 (PMC13166928; doi:10.1371/journal.pcbi.1013760)
Supplement: Attachment — Submitted filename: MALDI x MRSA Response to reviewers.docx [file pcbi.1013760.s007.docx]

**Response to Reviewers’ Comments**

Application of Machine Learning with MALDI-TOF MS for Rapid Differentiation between Methicillin-susceptible and Methicillin-resistant *Staphylococcus aureus*

Ms. No. PCOMPBIOL-D-25-01013

**Additional Editor Comments:**

Reviewer #1:


Reviewer #2: Address these comments


Reviewer #3: All 8 major issues raised by this reviewer should be addressed, especially those regarding the generalization capabilities of your tool

*Our Reply*

We are grateful for the constructive feedback provided by the reviewers. We have implemented changes and introduced external validation to address reviewer concerns.

**W**

**Reviewers' comments:**

**Response to Reviewer's Questions**

**Reviewer #1:**

This study investigates the potential application of novel machine learning techniques in conjunction with MALDI-TOF MS to develop a predictive model capable of rapidly distinguishing between methicillin-susceptible Staphylococcus aureus (MSSA) and methicillin-resistant Staphylococcus aureus (MRSA). Such differentiation can facilitate more appropriate antibiotic use, improve treatment outcomes, and support antibiotic stewardship efforts.

The top-performing models included a large-scale neural network (NN), the LightGBM gradient boosting framework (LGBM), and a weighted ensemble combining the NN and LGBM models. All three models demonstrated strong classification accuracies(>0.92), which were further enhanced through the application of a confidence threshold.

Upon thorough assessment of this manuscript, I am pleased to confirm that my concerns regarding scientific justification, methodological transparency, reproducibility, and data interpretation have been thoroughly addressed. The manuscript presents a well-structured and scientifically sound study, with text and figure clarity, proper citations, detailed methodological descriptions, and enhanced visual materials.

I recommend that the manuscript be accepted for publication.

*Our Reply*

We sincerely thank the reviewer for the positive feedback and for accepting our work for publication.

**Reviewer #2:**

This paper considers an application of ML to MSRA classification. The interest in the contents is likely moderate for readers of PLOS Comp Bio. The application of ML is reasonable, and the reference are up-to-date. The method appears competitive with many modern methods.

Reviewer Comment 2.1

My main concern is the use of confidence in this paper. The authors define confidence as = | − 0.5| + 0.5. This is not a statistical confidence measure, which the authors acknowledge by saying that it “can be interpreted as a confidence”. While it seems reasonable as a tool for calculating when to accept the prediction, based on Fig 2, I feel that the term confidence is likely not useful in this context. (However, the authors also define CIs for the models. Presumably, this is unrelated and calculated based on the confusion matrix itself.)
Having said this, some standardization of the “confidence” would be helpful. I believe the authors would benefit from using the literature on “machine learning with rejection”. For instance, this would allow the authors to standardize whether they refer to this condition as being “ignored” (line 422) or “rejected” (later on line 422). The authors may also benefit from the measures in the literature that discuss evaluation in the presence of rejection. An appropriate survey would be Hendrickx et al. 2024 ([https://link.springer.com/article/1 0.1007/s10994-024-06534-x](https://link.springer.com/article/1%200.1007/s10994-024-06534-x) ). I would encourage the authors to include some of these additional evaluation criteria (beyond what is shown in Fig 3) to help the readers understand the impact of rejecting, e.g., 20% of inputs.

*Our Reply*

We thank the reviewer for the constructive suggestions. In response, we have standardized the terminologies used throughout the manuscript in alignment with established literature. While we acknowledge that the proposed confidence formula is not a formal statistical measure, Figure 4 (lines 368-374) demonstrates that its output is strongly correlated with model accuracy. Therefore, we have opted not to retroactively introduce model calibration, as doing so may skew the results of prospective testing.

Regarding evaluation criteria, we appreciate the reference to the survey and have carefully considered its recommendations. Based on this, we regard the accuracy-rejection curve as the most appropriate and comprehensive evaluation tool for our study (i.e., lines 298-305; Figure 5, lines 386-395; Figure 6, lines 397-398).

In practice, rejected cases will not be reported in our laboratory, and we will instead await the results of culture and sensitivity testing, which remains the gold standard method (lines 382-384).

Comment 2.2

There is slight imbalance in the data set but the authors have appropriately used classification metrics that indicate that the class imbalance does not falsely inflate the results. Presumably, when the authors do their train-test and validation splits, they are done in a stratified manner. Please confirm or give data on the variation in data imbalance in the validation and prospective test sets.

*Our Reply*

Class distributions for the various datasets are presented in Supplementary Table 2 (line 689). The train-validation split was performed randomly, resulting in approximately equal class distributions. We experimented with applying class weights during loss calculation; however, the class imbalance was minimal, and most of our evaluation metrics are not sensitive to such imbalance. Consequently, there was no significant difference in performance with or without class weighting (lines 220-225).

Comment 2.3

The authors note on lines 115-117 “Limiting the scope of sample collection may potentially enhance model accuracy by focusing on features of local strains.” I believe this is intended to be described as an avenue for improving results, but I feel that readers may also want to consider that this offers an avenue for improving generalizability: that models that are insensitive to geographical location may be the goal of future work (or, on the other hand, that such models are unlikely to ever exist). Some more material here (or in another suitable location in the paper) would be beneficial.

*Our Reply*

We agree that this point warrants further discussion. In the revised version, we have introduced external testing to illustrate the challenges of generalizing models across different geographical regions. Our primary dataset originated in Hong Kong, while the newly added external validation was conducted using the data from Taiwan, a location geographically distinct from Hong Kong (lines 65-68; 138 (Figure 1); 173-180; 390-398; 451-468, 499-508).

Comment 2.4
Line 120 – reference is missing to Yu et al paper.

*Our Reply*

The previously missing reference has now been added to the manuscript (line 126).

**Reviewer #3:**

Summary: This paper applies machine learning to classify MRSA versus MSSA using MALDI-TOF spectra. The dataset is large (24,487 retrospective isolates and 2,975 prospective isolates from a single hospital), and models include a neural network, LightGBM, CatBoost, XGBoost, and an ensemble. Reported accuracies are 0.928–0.939 without thresholding, rising to >0.97 when ~20% of low-confidence cases are rejected. The study also incorporates SHAP-based feature analysis and a Streamlit web application for prospective testing. The dataset size and prospective validation are notable strengths. However, there are major concerns with reproducibility, generalizability, and several aspects of the methodology. At its current stage, the manuscript does not meet the journal’s standards.


**Major Critiques:**

Comment 3.1
Methods (p.17, Lines 305-311): All isolates are from a single hospital in Hong Kong. Without external validation on other sites or instruments, the results cannot be generalized. The authors should provide such data to verify their model’s generalizability.

*Our Reply*

We thank the reviewer for your valuable feedback. We would like to clarify that our dataset originated from two independent acute hospitals in Hong Kong—one with 2,000 acute beds and the other with 700 beds—collectively serving a population of approximately one million (line 160-162). To address the reviewer’s concern regarding the model’s generalizability, we conducted an external validation using MALDI-TOF data from the China Medical University Hospital in Taiwan, a location geographically distinct from Hong Kong (lines 65-68; 138 (Figure 1); 173-180; 390-398; 451-468, 499-508).

Comment 3.2

Discussion (p.16, Lines 283–290): The neural network requires hardware acceleration, while LightGBM is lightweight. The authors do not quantify model size or inference speed. Reporting accuracy versus inference time and memory is essential. Smaller NN architectures should be explored.

*Our Reply*

We acknowledge that LightGBM is lightweight and may be preferable in certain scenarios. During hyperparameter tuning, we explored smaller neural network (NN) architectures but observed inferior performance compared to LightGBM. Nonetheless, we consider NN a viable option, as it does not require hardware acceleration for inference. The Streamlit web interface used in our prospective testing was hosted on a server without GPU support, and preliminary testing showed that the NN model could predict 1,000 samples within 1.5 seconds, using less than 3GB of memory. We have included this point in the discussion to highlight the practical considerations of model deployment (lines 521-526).

Comment 3.3
3. Results (p.11, Lines 168–190): Accuracy gains rely on rejecting up to 20% of cases. The manuscript does not explain how rejected cases would be managed in practice or why rejection is preferable to other strategies. This undermines the clinical relevance of the approach.

*Our Reply*

In practice, rejected cases will not be reported in our laboratory, and we will instead await the results of culture and sensitivity testing, which remains the gold standard method (lines 382-384).

Comment 3.4

Methods (p.19, Lines 368–373): The same preprocessing pipeline is applied across all folds. Please clarify if spectra from the same patient or episode could appear in both train and test sets, and how deduplication was enforced. This is critical for unbiased validation.

*Our Reply*

We thank the reviewer for their valuable feedback. To ensure unbiased model validation, deduplication of spectra from the same patient was performed based on the hospital number within the same admission episode. As a result, MALDI-TOF spectra from the same patient or episode do not appear in both the training and validation sets (lines 192-196).

Comment 3.5

Results (p.12–13, Lines 195–238): SHAP identifies feature groups, but scatterplots show limited separation. Claims about consistent discriminatory power should be tempered. Consider reporting classification accuracy using only top SHAP features to strengthen the interpretation.

*Our Reply*

We agree with the reviewer’s observation and have noted in the manuscript that no single feature group consistently demonstrates strong discriminatory power. To further explore this, we trained a small multi-layer perceptron using each of the three feature groups individually. As reported in the manuscript, the model achieved an accuracy of approximately 0.8, highlighting the potential of combining feature groups for improved performance (lines 431-449).

Comment 3.6
Discussion (p.15, Lines 277–281; Supp. Fig. 3): The four-year analysis shows no clear decay, but this horizon is short. Bacterial strain turnover often requires longer monitoring. The authors should acknowledge this limitation.

*Our Reply*

We agree with the reviewer’s assessment and have incorporated this acknowledgement into the discussion section of the manuscript (lines 510-515).

Comment 3.7
Results (p.14, Lines 260–263; Table 2): The authors state sample size and confidence threshold are the biggest determinants of accuracy. However, Table 2 shows larger datasets with lower performance. This inconsistency suggests other factors matter.

*Our Reply*

The scope of this statement is limited to the dataset used in our study. Nonetheless, we acknowledge that the geographical distribution of samples is an important factor influencing model accuracy (lines 485-486).

Comment 3.8
Data and code availability (p.23, Lines 447–450): The datasets are not publicly available. Without this, the study is not reproducible.

*Our Reply*
The dataset used in this study has been anonymized and preprocessed and is now publicly available on Kaggle (lines 553-558).

**Minor Critiques:**

Comment 3.9

Abstract (p.3, Lines 33–59): The abstract is results-heavy. Streamline to emphasize clinical motivation, dataset size, and key findings.

*Our Reply*

We thank the reviewer for the suggestion and have implemented the recommended changes in the revised manuscript (lines 41-76).

Comment 3.10

Discussion (Table 2, p.14-15): Weis et al. (Nat Med 2022) is a major benchmark using the DRIAMS dataset (four hospitals, cross-site validation). This study is only briefly mentioned. A deeper comparison is needed, highlighting that Weis achieved generalization across sites, whereas this work is limited to one site.

*Our Reply*

We agree that the work by Weis et al. has been monumental in advancing this field. However, upon closer examination of their publicly available dataset, we observed a significant class imbalance and a relatively small proportion of samples pertaining specifically to MSSA/MRSA differentiation. While our model demonstrated superior performance in external testing compared to their cross-site validation, we believe this comparison is not entirely meaningful due to the lower MRSA prevalence during the period in which their data were collected.

Comment 3.11

Discussion (p.16, Lines 288–290): Report prediction times for NN, LightGBM, and ensemble models. Practical feasibility depends not just on accuracy but also speed.

*Our Reply*

We agree that prediction speed is an important consideration when assessing practical feasibility. However, during batch prediction, all top-performing models in our study required less than 5 milliseconds per prediction without hardware acceleration. Therefore, we believe that a detailed benchmarking of prediction speed is not necessary in this specific context.

Comment 3.12

Supp. Table 1 (p.29, Lines 577-579): How MRSA/MSSA ratios differ across specimen types is missing. Furthermore, clarify whether class weighting or balancing was used to avoid bias.

*Our Reply*

To enhance transparency, we have included a supplementary table detailing the MRSA/MSSA ratios in the training, validation, prospective, and external datasets (lines 689). Additionally, we have clarified that class weighting was not employed during model training due to the limited class imbalance and the use of evaluation metrics that are robust to such imbalance.

Comment 3.13

Methods (p.21, Lines 404–408): Clarify whether preprocessing and inference were frozen before prospective testing. Pipelines should be locked prior to deployment.

*Our Reply*

We have clarified in the manuscript that both preprocessing and inference procedures were fixed prior to prospective testing to ensure consistency and avoid data leakage (lines 271-273).

Comment 3.14

Confidence–accuracy plots (Fig. 3): Add error bars or confidence intervals to illustrate variability across folds.

*Our Reply*

We have added error bars to the plots to represent 95% confidence intervals across cross-validation folds, providing a clearer depiction of performance variability (Figure 4, lines 368-374).

Comment 3.15

SHAP stability: State whether top SHAP features are consistent across folds or vary between runs.

*Our Reply*

We have clarified that top SHAP features are consistent across folds (lines 412-419). SHAP spectral plots for each fold are available in the repository.

**Issue of Grammar, Spelling, Proofreading, etc.**

Comment 3.16

Several sentences are long and would be clearer if split (e.g., Introduction, p.6, Lines 87-91).

*Our Reply*

We thank the reviewer for the helpful suggestion and have implemented the corresponding changes in the revised manuscript.

Comment 3.17

Figure captions are short; expand them so each figure can be understood without referring to the main text.

*Our Reply*

We thank the reviewer for the helpful suggestion and have implemented the corresponding changes in the revised manuscript.
